# Home exposure to Arabian incense (bakhour) and asthma symptoms in children: a community survey in two regions in Oman

**DOI:** 10.1186/1471-2466-9-23

**Published:** 2009-05-19

**Authors:** Omar A Al-Rawas, Abdullah A Al-Maniri, Bazdawi M Al-Riyami

**Affiliations:** 1 Department of Medicine, College of Medicine and Health Science, Sultan Qaboos University, PO Box 35, Postal Code 123, Muscat, Sultanate of Oman; 2 Department of Family Medicine and Public Health, College of Medicine and Health Science, Sultan Qaboos University, PO Box: 35, Postal Code 123, Muscat, Sultanate of Oman

## Abstract

**Background:**

Incense burning has been reported to adversely affect respiratory health. The aim of this study was to explore whether exposure to bakhour contributes to the prevalence of asthma and/or triggers its symptoms in Omani children by comparing two Omani regions with different prevalence of asthma.

**Methods:**

A randomly selected sample of 10 years old schoolchildren were surveyed using an Arabic version of ISAAC Phase II questionnaires with the addition of questions concerning the use and effect of Arabian incense on asthma symptoms. Current asthma was defined as positive response to wheeze in the past 12 months or positive response to "ever had asthma" together with a positive response to exercise wheeze or night cough in the past 12 months. Simple and multivariable logistic regression analyses were performed to estimate the effect of bakhour exposure and other variables on current asthma diagnosis and parents' response to the question: "Does exposure to bakhour affect your child breathing?"

**Results:**

Of the 2441 surveyed children, 15.4% had current asthma. Bakhour use more than twice a week was three times more likely to affect child breathing compared to no bakhour use (adjusted OR 3.01; 95% CI 2.23–4.08) and this effect was 2.55 times higher in asthmatics (adjusted OR 2.55; 95% CI 1.97–3.31) compared to non-asthmatics. In addition, bakhour caused worsening of wheeze in 38% of the asthmatics, making it the fourth most common trigger factor after dust (49.2%), weather (47.6%) and respiratory tract infections (42.2%). However, there was no significant association between bakhour use and the prevalence of current asthma (adjusted OR 0.87; 95% CI 0.63–1.20).

**Conclusion:**

Arabian incense burning is a common trigger of wheezing among asthmatic children in Oman. However, it is not associated with the prevalence asthma.

## Background

Asthma is a worldwide major health problem with significant variations in its prevalence and severity in different parts of the world [1,2]. As a participating center in the International Study of Asthma and Allergies in Children (ISAAC), we have previously shown that asthma is common in Omani children with prevalence rates of 10.5% and 20.7% in 6–7 and 13–14 years old children respectively) [3]. In addition, Oman ranked among the top countries in ISAAC global ranking of symptoms taken to indicate severe asthma [2,3]. For example, the prevalence of waking one or more nights per week in the past 12 months in 6–7-year-old children was 3.5% (nearly 50% of all children with current wheeze), putting Oman in the top five countries for this severe symptom. Similarly, the 13–14-year-old group ranked among the top for the prevalence of severe asthma symptoms such as sleep disturbing wheeze and speech-limiting wheeze. We also found a significant variation in the prevalence of asthma diagnosis and symptoms among children from different geographical regions of the country [4,5].

As no information is available on the prevalence of asthma risk factors in Oman, we postulated that these findings may be due to differences in locally prevailing factors such as family history, sensitization to pollen and house dust mite, respiratory infections, and dietary habits, in addition to outdoor and indoor air pollutants including incense smoke. These factors may influence the pathogenesis and severity of asthma and require investigation not only to contribute to the understanding of asthma etiology, but also to plan measures for its control. [6]. In particular, it is important to explore the less characterized risk factors related to life style, culture and home environment which may be peculiar to different populations [7-9].

In Oman and other Gulf countries, Arabian incense (bakhour) is one of the common indoor smoke sources to which individuals are frequently exposed, and may be an important contributory factor to the observed high prevalence and severity of asthma in children of this region [10]. A wide variety of substances are used to produce bakhour including frankincense, aromatic wood, herbs, flowers, essential oils, and perfumes burned using charcoal burner [10]. Frankincense is a resin produced by oozing from incisions in the trunks of trees of the genus *Boswellia *that grow in the south of Oman (Dhofar) [11]. Other forms of incense are derived from sandalwood and are usually mixed with ingredients such as natural oils and perfumes [10].

Due to its slow and incomplete combustion, incense burning produces continuous smoke, generating pollutants such as toxic gases and chemicals particles including polycyclic aromatic hydrocarbons, carbon monoxide, benzene, and isoprene that easily accumulate indoors, especially under inadequate ventilation [12]. Exposure to incense smoke has been linked to several illnesses, including respiratory symptoms, asthma, elevated cord blood IgE levels, contact dermatitis and cancer [10,12-14]. In addition, it has been demonstrated that exposure to bakhour induced significant morphological changes in rats pneumocytes. [15]. The widespread use of bakhour in Oman, has prompted the need to explore whether exposure to bakhour contributes to the prevalence of asthma and/or triggers its symptoms in Omani children by comparing two Omani regions with different prevalence of asthma.

## Methods

### Subjects

Using ISAAC Phase II protocols, a target sample of 10 year old schoolchildren was randomly selected from a representative sample of public schools randomly chosen from two regions in Oman, Muscat and South Sharqiyah using stratified multi-stage sampling method. The selected two regions out of the ten regions of Oman were considered as potentially informative based on their different prevalence rates of asthma identified in ISAAC phase I, and the potential for differences in environmental exposures and asthma management patterns [4]. As the capital of Oman, Muscat population comes from most regions of the country, and the prevalence of asthma symptoms and diagnosis in Muscat resembles the national average, whereas South Sharqiyah (Eastern) region has the highest prevalence rates of all asthma symptoms [4]. For example the prevalence of self reported asthma in 13–14 year old children was 20.7% in Oman (national average), 19.1% in Muscat and 30.6% in South Sharqiyah [4]. The sample frame of the survey was a list of public schools provided by the Ministry of Education indicating the district and town, number of schools and the numbers of children in primary grades V and VI in these two regions. Ethical approval of the study protocol was obtained from the Ministry of Health and the Ministry of Education as well as the Research and Ethics Committee of the College of Medicine and Health Sciences, Sultan Qaboos University.

### The Questionnaire

The survey used an Arabic version of ISAAC Phase II core and supplementary questionnaires developed according to ISAAC translation protocol [16]. The core questionnaires consisted of two parts: demographic characteristics and ISAAC core questionnaire on wheezing, rhinitis and eczema. The supplementary questionnaires consisted of three parts: a) additional respiratory questions regarding cough with phlegm and wheeze with breathlessness, b) questions on disease management for asthma rhinitis and eczema and c) questions on risk factors. The risk factors questionnaire consists of four sections: a) early days of life, b) diseases and immunization, c) home conditions and d) child behavior (exercise and food). Based on our hypothesis that Arabian incense (bakhour) may be a locally relevant indoor risk factor for asthma, we added five questions addressing the frequency, and pattern of bakhour usage in addition to its effect on children as follows: 1) Do you use bakhour at your home? 2) If yes, how many times per week? "3 options: a) never or rarely, b) 1–2 times, and c) more than twice". 3) Do you use bakhour in the presence of the child? 4) Do you use bakhour in the child room? And 5) does exposure to bakhour affect your child breathing? The validity of these questions was assess by face validity by the research team. Chronabch's alpha, as a measure of reliability, of these questions was 0.64. Bakhour use was also added to the ISAAC Phase II supplementary questions (Module 2.1) list of responses to the question "in the last 12 months what has made your child's wheezing worse?" The list contained the following possible asthma triggers: weather, pollen, emotions, fumes, dust, pets, wool clothing, colds or flu, cigarette smoke, foods and drinks, soaps and sprays in addition to bakhour. All questionnaires were distributed through the schools to children in April 2001. They were completed by parents and returned to schools with the same week.

### Outcome Measures

The primary outcome variables were the prevalence of current asthma diagnosis and parents' response to the question: "Does exposure to bakhour affect your child breathing?" In ISAAC studies, current asthma is defined by wheeze in the past 12 months (current wheeze) [17]. However, children with positive response to "ever had asthma?" together with positive responses to other core asthma symptoms in the past 12 months (exercise wheeze and/or night cough not related to cold) can also be considered as having current asthma. Thus, for the purpose of this analysis, current asthma was defined as positive response to wheeze in the past 12 months or positive response to "ever had asthma" together with a positive response to exercise wheeze or night cough in the past 12 months. Secondary outcomes include difference between the two surveyed regions (Muscat and South Sharqiyah) in the prevalence of asthma symptoms and trigger factors of wheeze in children with asthma.

### Statistical Analysis

Data were collected and entered according to the ISAAC protocol and were analyzed using SPSS package for Window version 10 (SPSS Inc. Chicago, IL, USA). Frequency and percentage of different variables were computed. Comparisons between Muscat and South Sharqiyah were performed using the Chi-squared test. Simple and multivariable logistic regression analyses were performed to estimate the effect of bakhour use and other potential risk factors on the primary outcome variables (current asthma and parents reporting that the child breathing is affected by bakhour). A p value of = 0.05 was considered as statistically significant and 95% Confidence Intervals (CI) of the estimates were computed.

## Results

A total of 2535 questionnaires were distributed and 2441 (1241 from Muscat) were returned and available for analysis (96% response rate; 95% in Muscat and 97% in South Sharqiyah). The mean age (SD) of children was 10.7 (0.9) years, and was slightly higher in South Sharqiyah; 10.8 (0.9) years compared to Muscat; 10.6 (0.9) years (p < 0.001). The gender distribution was similar in the two regions (males constituted 54.3% and 51.3% in Muscat and Sharqiyah respectively, p = 0.141). The prevalence of current asthma diagnosis in the study sample was 15.4%.

Table 1 shows the frequency (%) of parents' responses to core asthma symptoms, additional respiratory symptoms and bakhour use information compared by region. The prevalence of core asthma symptoms, asthma diagnoses (both current asthma and ever had asthma) and other respiratory symptoms were all significantly higher in Sharqiyah, except symptoms of severe asthma, which were either similar in the two regions (more than three episodes of wheeze and speech limiting wheeze) or higher in Muscat (sleep disturbance one night or more per week). History of atopy was also higher in Sharqiyah. However, the difference was only significant for child history of hay fever and father history of atopy. The use of bakhour at home was equally common in both regions. However, its use more than twice per week was significantly higher in Sharqiyah. Although, parents from Sharqiyah reported less frequent use of bakhour in the presence of the child and in the child's room, they reported higher number of children affected by bakhour compared to Muscat.

**Table 1:** Comparison of parents' responses to core asthma symptoms, additional respiratory symptoms and bakhour use by region

	Muscat n = 1241	S. Sharqiyah n = 1200	*P-*value
Core asthma symptoms			
12-month prevalence of:			
▪ Any wheeze	9.3	15.8	<0.001
▪ Exercise induced wheeze	6.4	10.1	0.001
▪ Night cough in absence of cold	17.2	23.4	<0.001
▪ 1–3 wheezing episodes	4.8	10.4	<0.001
▪ More than 3 wheezing episodes	2.1	2.3	0.689
▪ Sleep disturbance one night or more/week	1.5	0.5	0.034
▪ Speech limiting wheeze	1.5	1.2	0.441
Ever had asthma	12.1	21.3	<0.001
Current asthma	10.7	20.3	<0.001

Additional Respiratory symptoms			
12-month prevalence of:			
▪ Wheeze during cold or flu	6.5	15.2	<0.001
▪ Cough and phlegm with colds	18.0	29.2	<0.001
▪ Cough and phlegm without colds	8.0	14.8	<0.001
Ever woken up with shortness of breath	7.9	13.8	<0.001
Ever woke up with chest tightness	5.2	8.6	0.001

Atopy			
▪ Ever had hay fever	9.3	15.3	<0.001
▪ Ever had eczema	10.7	12.8	0.122
▪ Father has asthma, hay fever or eczema	12.9	16.9	0.033
▪ Mother has asthma, hay fever or eczema	16.9	19.8	0.071

Bakhour use at home	91.5	91.0	0.689

Frequency of use per week			
▪ Never or rarely	19.0	19.0	1.000
▪ 1–2 times/week	27.2	17.8	<0.001
▪ >2 times/week	53.8	63.2	<0.001

Bakhour use in presence of child	60.5	54.8	<0.001

Bakhour use in the child room	61.2	54.3	0.004

Child affected by bakhour	19.3	29.5	0.001

Table 2 presents crude and adjusted Odds Ratio (OR) with 95% CI of the effect of current asthma diagnosis and other potential confounding variables on parents reporting that the "child breathing is affected by bakhour". In both simple and multivariable regression analysis, current asthma, frequency of bakhour use, bakhour use in presence of the child, bakhour use in child room, gender of the child, region of residence and parent level of education were all associated with reporting that child breathing is affected by bakhour. Parents of children with current asthma were more likely to report that their children were affected by bakhour compared to parents of children with no current asthma (adjusted OR = 2.55; 95% CI 1.97–3.31). Parents who reported frequent use of bakhour at home (more than twice per week) were more likely to report that their children were affected by bakhour compared to parents who never or rarely used bakhour (adjusted OR = 3.01, 95%CI 2.23–4.08). However, parents who reported that the child was affected by bakhour were less likely to report bakhour use in the presence of the child or in the child room. In addition, parents of girls and parents from Sharqiyah were more likely to report that their children were affected by bakhour compared to parents of boys and parents from Muscat, respectively. There was no significant association between current parental smoking and reporting the effect of bakhour use on the child.

**Table 2:** Crude and adjusted Odds Ratio (OR) with 95% Confidence Intervals (CI) of the effect of current asthma and other variables on reporting that the child is affected by exposure to bakhour using logistic regression analysis.

	Number (%) of total sample	(%) affected by bakhour	Crude OR (95% CI)	Adjusted OR# (95% CI)
Current asthma				
▪ No	2065 (84.6)	43.6	1.00	1.00
▪ Yes	376 (15.4)	56.4	2.32 (1.84–2.93)*	2.55 (1.97–3.31)*

Bakhour use per week				
▪ Never or rarely	463 (19.0)	19.7	1.00	1.00
▪ 1–2 times/week	549 (22.5)	22.8	1.21 (0.89–1.63)	2.28 (1.63–3.21)*
▪ >2 times/week	1423 (58.4)	26.6	1.48 (1.14–1.91)*	3.01 (2.23–4.06)*

Bakhour use in presence of child				
▪ No	1032 (42.3)	35.1	1.00	1.00
▪ Yes	1409 (57.7)	16.5	0.37 (0.30–0.44)*	0.51 (0.40–0.65)*

Bakhour use in the child room				
▪ No	1030 (42.2)	36.4	1.00	1.00
▪ Yes	1411 (57.8)	15.5	0.32 (0.27–0.38)*	0.36 (0.28–0.46)*

Gender				
▪ Male	1290 (52.8)	21.9	1.00	1.00
▪ Female	1151 (47.2)	24.3	1.33 (1.04–1.60)*	1.31 (1.07–1.61)*

Region				
▪ Muscat	1241 (50.8)	19.3	1.00	1.00
▪ Sharqiyah	1200 (49.2)	29.5	1.75 (1.45–2.10)*	1.31 (1.07–1.62)*

Father education				
▪ No School education	973 (41.1)	30.2	1.00	1.00
▪ Didn't complete High School	905 (38.2)	22.8	0.68 (0.55–0.83)*	0.79 (0.62–1.00)
▪ Completed High School	492 (20.8)	15.4	0.42 (0.32–0.55)*	0.65 (0.45-.92)*

Mother education				
▪ No School education	1328 (55.3)	27.7	1.00	1.00
▪ Didn't complete High School	829 (34.5)	21.6	0.72 (0.59–0.88)*	0.86 (0.67–1.10)
▪ Completed High School	244 (10.2)	13.9	0.42 (0.29–0.61)*	0.68 (0.43–1.07)*

Any parent smoking				
▪ No	1860 (76.2)	24.4	1.00	1.00
▪ Yes	581 (23.8)	24.3	1.00 (0.80–1.23)	1.02 (0.80–1.31)

*Statistically significant at p-value < 0.05

# adjusted for all variables listed in the table.

Table 3 shows the results of simple and multivariate logistic regression analysis with having current asthma as dependent and different risk factors, including bakhour use as independent variables. Children from Sharqiyah were reported to have significantly higher prevalence of current asthma compared to children from Muscat (adjusted OR = 1.79, 95% CI 1.38–2.32). In addition, parent history of atopy, parent history of current smoking, use of air conditioning and use of loose carpets in child room were associated with current asthma. Although, bakhour use of 1–2 times per week was associated with lower prevalence of current asthma, in simple regression analysis (OR = 0.59; 95% CI 0.42–0.84), this association was not significant after adjusting for all other risk factors (adjusted OR = 0.68; 95% CI 0.46–1.01). Table 4 shows that there was no significant difference between bakhour users and non users in the prevalence of any of asthma core symptoms or any of the additional respiratory symptoms.

**Table 3:** Crude and adjusted Odds Ratio (OR) with 95% Confidence Intervals (CI) for home related risk factors for asthma using logistic regression analysis

	Number (%) of total sample	Current asthma prevalence (%)	Crude OR (95% CI)	Adjusted OR# (95% CI)
Gender				
▪ Male	1290 (52.8)	15.0	1.00	1.00
▪ Female	1151 (47.2)	15.9	1.08 (0.86–1.34)	1.08 (0.85–1.37)

Region				
▪ Muscat	1241 (50.8)	10.7	1.00	1.00
▪ Sharqiyah	1200 (49.2)	20.3	2.12 (1.68–2.66)*	1.79 (1.38–2.32)*

Atopy in parents				
▪ None	1836 (75.2)	11.3	1.00	1.00
▪ One parent	412 (16.9)	27.8	2.61 (2.900–340)*	2.48 (1.86–3.29)*
▪ Both parents	193 (7.9)	33.7	3.98 (2.85–5.54)*	3.23 (2.425–4.63)*

Father education				
▪ No School education	973 (41.1)	17.1	1.00	1.00
▪ Didn't complete High School	905 (38.2)	15.5	0.89 (0.70–1.14)	1.18 (0.89–1.58)
▪ Completed High School	492 (20.8)	10.8	0.59 (0.42–0.82)*	1.16 0.77–1.75)

Mother education				
▪ No School education	1328 (55.3)	17.3	1.00	1.00
▪ Didn't complete High School	829 (34.5)	14.4	0.80 (0.63–1.02)	0.95 (0.72–1.27)
▪ Completed High School	244 (10.2)	8.6	0.45 (0.28–0.72)*	0.62 (0.36–1.07)

Any parent smoking				
▪ No	1860 (76.2)	13.9	1.00	1.00
▪ Yes	581 (23.8)	20.3	1.66 (1.24–2.01)*	1.59 (1.22–2.07)*

Bakhour use				
▪ Never or rarely	463 (19.0)	18.8	1.00	1.00
▪ 1–2 times/week	549 (22.5)	12.6	0.59 (0.42–0.84)*	0.68 (0.46–1.01)
▪ >2 times/week	1423 (58.4)	15.7	0.80 (0.61–1.06)	0.87 (0.63–1.20)

Use of gas cooking				
▪ No	547 (22.4)	14.3	1.00	1.00
▪ Yes	1894 (77.6)	15.7	1.12 (0.86–1.47)	1.13 (0.83–1.55)

Air conditions				
▪ No	287 (11.8)	25.8	1.00	1.00
▪ Yes	2154 (88.2)	14.0	0.47 (0.51–0.63)*	0.56 (0.40–0.80)*

Damp spots on house walls				
▪ No	2218 (90.9)	14.7	1.00	1.00
▪ Yes	223 (9.1)	22.9	1.72 (1.24–2.41)*	1.47 (1.01–2.12) *

Use of carpet				
▪ None	343 (14.1)	15.2	1.00	1.00
▪ Fitted carpet	1549 (63.5)	12.5	0.80 (0.57–1.12)	0.80 (0.56–1.16)
▪ Loose carpet	131 (5.4)	29.0	2.29 (1.42–3.69)*	1.99 (1.11–3.24)*
▪ Fitted and loose carpets	418 (17.1)	22.2	1.76 (1.10–2.33)*	1.25 (0.81–1.94)

Feather pillow				
▪ No	2177 (89.2)	14.8	1.00	1.00
▪ Yes	264 (10.8)	20.1	1.44 (1.04–2.33)*	1.14 (0.80–1.64)

Cat ownership				
▪ No	1785 (73.1)	14.3	1.00	1.00
▪ Yes	656 (26.9)	18.3	1.34 (1.05–1.70)*	1.18 (0.90–1.52)

*Statistically significant at p-value 0.05

# adjusted for all variables listed in the table

**Table 4:** The percentage of core asthma symptoms and other respiratory symptoms in children compared by bakhour use

	Bakhour use		
		
	Yes (n = 2227) %	No (214) %	*P-*value
Core asthma symptoms			
12-month prevalence of:			
▪ Any wheeze	13.1	16.8	0.055
▪ Exercise induced wheeze	7.9	11.2	0.097
▪ Night cough in absence of cold	20.5	18.2	0.434
▪ More than 3 wheezing episodes	2.4	1.9	0.721
▪ Sleep disturbance one night or more/week	1.1	0.9	0.846
▪ Speech limiting wheeze	1.4	0.9	0.580
Ever had asthma	16.8	16.8	0.992

Additional Respiratory symptoms			
12-month prevalence of:			
▪ Wheeze during cold or flu	10.9	9.8	0.621
▪ Cough and phlegm with colds	23.9	19.2	0.119
▪ Cough and phlegm without colds	11.3	7.0	0.054
Ever woken up with shortness of breath	10.8	10.7	0.990
Ever woke up with chest tightness	7.0	5.1	0.292

**Figure 1:**
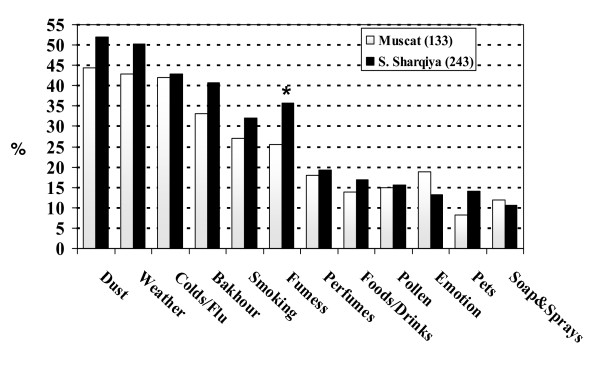
**Frequency of parent-reported wheeze trigger factors in children with current asthma (n = 376) stratified by region**. * significant difference between the two regions (p value = 0.042).

Figure 1 shows the frequency of triggers of wheeze among children with current asthma stratified by region. Although, there was a trend for higher frequency of various wheeze triggers in Sharqiyah region, the difference was not statistically significant except for fumes (p = 0.042). Bakhour was reported as a cause of worsening wheeze in 33.1% and 40.7% (p = 0.144) of children with current asthma in Muscat and Sharqiyah respectively, making it the fourth most common factor for worsening wheeze after dust, weather and viral upper respiratory tract infections in both regions.

## Discussion

This study builds on our prior work as participating center in the ISAAC phases one and three by confirming the previous findings in another age group (10 year old children) using ISAAC phase II protocols. Consistent with our previous finding in ISAAC phase one (1995) and phase three (2001) surveys conducted on 6–7 and 13–14 year old children, the prevalence rates of asthma symptoms and diagnosis in these 10 year old children were significantly higher in South Sharqiyah region [4,5]. More importantly, this study explores the role of the Arabian incense burning "bakhour" which is commonly used in Oman and other Gulf countries, as a potential indoor environmental risk factor for asthma symptoms. In this study, we evaluated the relationship between the frequency of bakhour use and asthma symptoms and its interaction with other potentially relevant factors.

After controlling for potential confounding factors in multivariate analysis (Table 2), we found a significant dose-response association between the frequency of bakhour use and its effect on these children breathing especially asthmatics. Parents who reported regular use (more than twice per week) of bakhour at home were three times more likely to report that their child breathing was affected by bakhour compared to parents who rarely used bakhour, and this was more 2.5 times higher in children with current asthma. The effect of bakhour was also significantly higher in girls and in children from Sharqiyah. In addition, bakhour was reported (Figure 1) as a cause of worsening wheeze in nearly 40% of children with current asthma, making it the fourth most common precipitating factor of wheeze (after dust, weather and viral upper respiratory tract infections). However, we found no significant association between bakhour use and the prevalence of current asthma (Table 3). There was also no significant difference between bakhour users and non users in the prevalence of any of the surveyed respiratory symptoms (Table 4). These findings suggest that bakhour is a common trigger factor of respiratory symptoms among Omani school children especially asthmatics and females. However, it is not be associated with higher prevalence of current asthma.

As there is no data on the contribution of incense burning to indoor air pollution in Oman, it is reasonable to assume that a higher frequency of incense burning produces a higher concentration of incense smoke pollutants. In general, most people spend approximately 90% of their time indoors [18]. Due to the very hot weather in Oman, it is expected the time spent indoors to be even higher especially for women and children. This may explain the observed greater susceptibility of girls to the effect of bakhour. The cause of the higher effect of bakhour on children from Sharqiyah region is not clear. Possible explanations include greater susceptibility [7], presence of other confounding factors not controlled for in the present analysis [5] and/or the difference in the composition of bakhour which is known to vary between regions within Oman. Further studies are necessary to determine the factors which influence the susceptibility to bakhour.

In the three studies that looked at the relationship between bakhour and asthma in the Arabian Gulf region, exposure to different types of Arabian incense was also reported as a significant precipitating factor of asthma symptoms in children [10,13,14]. However, none of these studies evaluated the relationship between bakhour and the prevalence of asthma. In Qatar, a case control study, found history of exposure to bakhour in 80% of asthmatic children compared to 66% of normal controls, [10] and in another study, bakhour was reported as precipitating factor of asthma symptoms in 19% of children with asthma [13]. Exposure to bakhour was also reported as trigger for asthma in 26% of 135 consecutive asthmatics attending a pediatric asthma clinic in a tertiary referral centre in Kuwait [14].

Several other studies from Asian populations, where different types of incense are burnt for religious purposes, reported significant associations between exposure to incense smoke and respiratory symptoms [12,19,20]. In contrast, one large survey of school children, showed incense burning at home was negatively associated with the prevalence of asthma [21]. The authors suggested that incense use might have been decreased in families with asthmatic children. In another study of 346 primary school children, there was no association between exposure to incense burning and respiratory symptoms [22]. Likewise, a case control study of 50 non smoking Saudi women with chronic obstructive pulmonary disease, reported no significant difference in the use of bakhour between cases and control [23].

Similarly our study did not find any significant association between bakhour use and the prevalence of current asthma. In fact there was an inclination towards a negative association which was significant in simple regression analysis, but disappeared after adjusting for other factors in the multivariate analysis. There was also no significant difference between bakhour users and non users in the prevalence of core asthma symptoms or any of the surveyed respiratory symptoms. Since our study is a cross-sectional, it is possible that families with asthmatic children avoid the use of bakhour after learning that bakhour might worsen asthma symptoms. Such avoidance behavior may influence the relationship between the reported prevalence of asthma and exposure to bakhour leading to underestimation or even reversal of any association. In our study, this was supported by the finding that parent who reported that their children were affected by bakhour exposure were less likely to report its use in the presence of child or in the child room.

The mechanisms by which bakhour provokes respiratory symptoms are unknown. People who are exposed to incense smoke inhale a complex mixture that contains particulate matter, gas products and many organic compounds, making it difficult to single out the health effects contributed by a certain component in the smoke. Furthermore, there appears to no standardized constituents of different types of bakhour even within the same country [12]. Our study has its limitations. The use of symptoms questionnaire to distinguish asthmatics and non asthmatics has potential problems arising from subjective symptom recognition and recall. In addition, as they were new to the Arabic version of ISAAC phase II, bakhour questions needed to have more scrutinized measures of validity and reliability. In ISAAC studies, current asthma is defined by wheeze in the past 12 months (current wheeze), regardless of the response to "ever had asthma?" question [16]. In addition, we included children with positive response to "ever had asthma?" together with positive responses to exercise wheeze and/or night cough not related to cold in the past 12 months in current asthma category. Like any cross-sectional study, this report lacks clear temporal association between exposure and its effect. We tried to overcome this problem with the question "does exposure to bakhour affect your child breathing?" In addition, the finding of bakhour exposure as one of the most frequent triggers of wheeze in our children suggests a temporal association. Finally, because of our primary focus, only few of asthma risk factors were evaluated.

## Conclusion

This study demonstrated that burning of Arabian incense (bakhour) is common practice in Omani households and is an important trigger of wheezing among children with asthma. However, there was no association between bakhour use and the prevalence asthma in these children. To obtain more conclusive information, detailed studies are needed to address the various characteristics of incense burning practice and its respiratory effects, including: identifying the most common types, their compositions and the temporal association with respiratory symptoms, in addition to lung function measurements in high exposure individuals. Furthermore, the effect of incense smoke on asthma and the respiratory system and its mechanism needs further studied in animal models. As bakhour use is very common in most Arab communities, our findings have important public health implications. With the currently available evidence, it is important to raise public awareness about the potential harmful effects of incense burning in order to take steps to reduce exposure such as reducing the frequency and duration of incense burning, keeping the room well ventilated when burning incense and avoiding such practice in the presence of children and susceptible individuals.

## Competing interests

The authors declare that they have no competing interests.

## Authors' contributions

OAA is the principal investigator and corresponding author.He wasinvolved in the design of the study, data collection and analysis, interpretation of the results and drafting the manuscript. AAA is anepidemiologist, participated in data analysis and manuscript writing. BMAwas involved in the design of the study, data collection and drafting the manuscript. All authors read and approved the final manuscript.

## Pre-publication history

The pre-publication history for this paper can be accessed here:


